# Molecular Analysis and Conformational Dynamics of Human MC4R Disease-Causing Mutations

**DOI:** 10.3390/molecules27134037

**Published:** 2022-06-23

**Authors:** Munazza Tamkeen Fatima, Zeyaul Islam, Prasanna R. Kolatkar, Ammira Sarah Al-Shabeeb Akil

**Affiliations:** 1 Department of Human Genetics, Translational Medicine Division, Research Branch, Sidra Medicine, Doha 26999, Qatar; mtamkeen-la@sidra.org; 2 Diabetes Research Center (DRC), Qatar Biomedical Research Institute (QBRI), Hamad Bin Khalifa University (HBKU), Qatar Foundation, Doha 34110, Qatar; zislam@hbku.edu.qa

**Keywords:** MC4R, G protein-coupled transporter (GPCR), obesity, pathological variants, mutational analysis, simulation

## Abstract

Obesity is a chronic disease with increasing cases among children and adolescents. Melanocortin 4 receptor (MC4R) is a G protein-coupled transporter involved in solute transport, enabling it to maintain cellular homeostasis. MC4R mutations are associated with early-onset severe obesity, and the identification of potential pathological variants is crucial for the clinical management of patients with obesity. A number of mutations have been reported in MC4R that are responsible for causing obesity and related complications. Delineating these mutations and analyzing their effect on MC4R’s structure will help in the clinical intervention of the disease condition as well as designing potential drugs against it. Sequence-based pathogenicity and structure-based protein stability analyses were conducted on naturally occurring variants. We used computational tools to analyze the conservation of these mutations on MC4R’s structure to map the structural variations. Detailed structural analyses were carried out for the active site mutations (i.e., D122N, D126Y, and S188L) and their influence on the binding of calcium and the agonist or antagonist. We performed molecular dynamics (MD) simulations of the wild-type and selected mutations to delineate the conformational changes, which provided us with possible reasons for MC4R’s instability in these mutations. This study provides insight into the potential direction toward understanding the molecular basis of MC4R dysfunction in disease progression and obesity.

## 1. Introduction

Obesity forms a complex multifactorial disease, with a global prevalence of 12%. Studies have suggested a strong genetic influence affecting obesity, with the melanocortin 4 receptor (MC4R) being one of the most critical and widely investigated so far. It is a member of the G protein-coupled receptor (GPCR) family, a major drug target accounting for 30% of FDA-approved medicine [1,2]. MC4R is expressed in the hypothalamus (i.e., paraventricular nucleus), and it is a key component of the leptin–melanocortin pathway [3]. The MC4R is activated by proopiomelanocortin (POMC)-derived polypeptides: α- and β-melanocyte-stimulating hormone (MSH), released by the post-translational processing of POMC. Conversely it is blocked by agouti-related peptide (AgRP) expressed in the AgRP/neuropeptide Y(NPY) neurons in the arcuate nucleus [4]. The function of these neurons is regulated via signals received from adipose tissue, precisely by leptin via the leptin receptor in the case of food and energy metabolism. MSH activates the MC4R and catalyzes the exchange of GDP for GTP on the stimulatory G protein (Gs), resulting in activation of adenylyl cyclase (AC) and generation of intracellular cAMP. cAMP activates protein kinase A(PKA) or MAPK signaling (via ERK1/2 phosphorylation) [5,6]. However, more recently, the role of other crucial pathways has been suggested [2,7]. Reports on potential involvement of β-arrestin and MC4R signaling has broadened the overall complexity [6]. Activation of MC4R via regulation of calcium ions and potassium channels has also been reported to be extremely crucial [8].

More than 170 variants of MC4R have been reported to be linked to hyperphagia and early-onset obesity and, in contrast, several variants alleviate the pathology by lowering BMI and other obesity-associated conditions, referred to as gain of function (GoF) mutations [9]. Homozygous variants are rare and afflict earlier/severe obesity [10]. Heterozygous mutations in MC4R alone, however, are implicated in 6% of early-onset severe obesity in the adult population alone [11]. These mutations result in partial or complete loss-of-function as well as GoF, depending on the nature or function of the mutated receptors as tested in vitro [12]. Conflicting reports regarding the prevalence and phenotypic effects of these variants in different cohorts is also a concern [9]. 

MC4R is a structurally divergent GPCR and exerts a gene dosage effect seldom seen in other GPCRs [13]. It is regulated by an endogenous agonist peptide, α-melanocyte-stimulating hormone (α-MSH), as well as by endogenous antagonists such as the agouti-related protein [14]. MC4R is an important drug target due to the prevalence of mutations causing monogenic obesity and other forms of obesity [15,16], but the unusual nature of MC4R has hampered the development and application of drugs that target MC4R and demand detailed insight into the molecular mechanism of MC4R and associated mutations. We consolidated the effects of mutations on the pathogenicity and protein stability using computational techniques. We analyzed the binding pocket of MC4R complex structures and investigated the effects of crucial mutations on metal (calcium) and agonist/antagonist binding. To gain insight into the structural changes caused by MC4R mutations, we applied molecular dynamics (MD) simulations to predict the effect of binding pocket mutations on the stability and flexibility of MC4R in comparison to wild type. Our results highlight potential differences due to the presence of mutations and their implications on the molecular mechanism. These studies open a path for initiating functional studies and future applications in personalized medicine.

## 2. Material and Methods

### 2.1. Sequence and Structural Data Information

The protein sequence of MC4R and its related family members were retrieved from UniProt (Universal Protein Resource, URL: https://www.uniprot.org/). UniProt is a common collection of protein sequences and contains millions of protein annotations across all branches of life [17]. Naturally occurring mutations were taken from the Ensembl (https://asia.ensembl.org/index.html) [18] database and manually curated to add the missing recently reported mutations. The structural coordinates of human MC4R (in complex with calcium, agonist, and antagonist) solved by X-ray crystallography, as well as by cryogenic-electron microscopy (cryo-EM), were retrieved from the RCSB Protein Data Bank (PDB, URL: https://www.rcsb.org).

### 2.2. Sequence-Based Prediction of Deleterious Mutations

In the present study, various web-based tools were utilized to predict the functional impact and pathogenic nature of nonsynonymous single-nucleotide polymorphisms (nsSNPs) in MC4R. These included Sorting Intolerant from Tolerant (SIFT), Polymorphism phenotyping-2 (Polyphen-2), and Mutant accessor (Mut_accessor). These tools classified the mutations as having either deleterious or non-deleterious effects. The SIFT algorithm (https://sift.bii.a-star.edu.sg/) is based on a sequence homology approach and makes use of the physical properties of the amino acid residues to predict whether a mutation is tolerable or deleterious. A SIFT score of <0.05 is considered intolerable [19]. Polyphen-2 (http://genetics.bwh.harvard.edu/pph2/index.shtml) predicts the impact of nonsynonymous mutations/amino acid substitution through an evolutionary sequence and structure-based approach. It calculates a position-specific independent count (PSIC) score for the mutation in question and compares the difference with the normal protein [20]. A PSIC score of >0.09 is deleterious. Mut_accessor (http://mutationassessor.org/r3/) predicts the impact of a mutation on the functionality of the protein. It is based on multiple sequence alignment as well as on the evolutionary conservation of amino acid residues [21]. It further classifies the deleterious mutations as having neutral, low, or median effects. An FI score was generated for each nonsynonymous mutation, and a value above 2 was measured as deleterious.

### 2.3. Structure-Based Predictors to Investigate Protein Stability/Destabilizing Mutations

We used five computational tools for a predictive in silico analysis of the impact of the mutations on the structure and function of MC4R. These included a mutation cut off scanning matrix (mCSM), Site-Directed Mutator2 (SDM2), DUET, iMUT2.0, and MAE. These are efficient and versatile tools for predicting disease-associated mutations in a wide range of proteins. mCSM (http://structure.bioc.cam.ac.uk/mcsm) utilizes a graph-based approach to envisage the protein destabilizing effect of a nonsynonymous mutation [22]. The distance pattern between atoms is used to represent the environment of the amino acids/protein to train predictive models utilizing thermodynamic datasets from the ProTherm, ProNIT [23], and SKEMPI [24] databases. The impact of the mutation on each amino acid is associated with the atomic distance patterns adjoining that residue. An mCSM score (ddG) < 0 indicates a destabilizing/altering effect of the mutation on protein function. 

SDM2 is a tool (http://marid.bioc.cam.ac.uk/sdm2) that calculates the alterations in protein stability by comparing the mutant protein to the wild type, utilizing the PDB coordinate files and environment-specific amino acid substitution tables (ESSTs) [25] and compares their thermal stability (mutant vs. wild type). New structural parameters, including the length of the residue and packing density, have also been included for the calculation of ESSTs. DUET (http://structure.bioc.cam.ac.uk/duet) was developed by integrating the features of mCSM and SDM to improve the overall utility [26]. The combining features include the secondary structure and solvent accessibility from SDM and pharmacophore vectors from mCSM. It utilizes support-vector-machine-based supervised learning, joining the scores of mCSM and SDM to provide the combined delta delta G values [26]. iMUT2.0: I-Mutant (https://folding.biofold.org/i-mutant/i-mutant2.0.html) predicts the changes in stability upon a mutation in the protein sequence or protein structure. It is trained on a ProTherm derived dataset and provides the change in free energy values for either all possible mutations of a particular residue or only for a specific mutation [27]. MAESTROweb (https://biwww.che.sbg.ac.at/) evaluates the effect of multiple mutations at the same position as well as those occurring at different positions [28]. A MAESTRO score < 0 predicts a destabilizing effect of the mutation.

### 2.4. Conservation of Amino Acid Positions in the MC4R Sequence

Multiple sequence alignment was performed using Clustal Omega (https://www.ebi.ac.uk/Tools/msa/clustalo/) (using seeded guide trees and HMM profile–profile techniques to generate alignments) [29], and annotation was conducted by the multiple alignment web server (http://www.bioinformatics.org/sms/multi_align.html). We used the ConSurf server to analyze the evolutionary conservation of amino acid positions in MC4R using an empirical Bayesian inference [30]. ConSurf provides the evolutionary conservation profile of amino acids by identifying the conserved position using multiple sequence alignment and estimates the evolutionary conservation rate using empirical Bayesian. The conservation scores were divided into a discrete scale of nine grades for visualization, from the most variable positions (i.e., grade 1), through intermediately conserved positions (i.e., grade 5), to the most conserved positions (i.e., grade 9) [30]. This tool also divides the amino acid residues based on solvent accessibility into four categories: e—an exposed residue according to the neural network algorithm; b—a buried residue according to the neural network algorithm; f—a predicted functional residue (highly conserved and exposed); s—a predicted structural residue (highly conserved and buried).

### 2.5. MD Simulation of MC4R Complex Structures

The molecular dynamics simulation for the complex MC4R and its mutant were run with YASARA STRUCTURE version 21.12.19. [31]. YASARA builds a membrane, embeds the protein, and runs a 250 ps equilibration simulation, during which the membrane is artificially stabilized while it adapts to the protein. The MC4R and its mutant form simulation were set-up automatically by first scanning the protein for exposed transmembrane helices and were run with periodic boundaries. The simulation was run for 200 nanoseconds using the AMBER14 force field [32] along with Lipid17 [33]/GAFF [34]/AM1BCC [35] force field parameters for nonstandard residues. The protein sidechain, pKas, was predicted [36]; protonation states were assigned according to pH 7.4; the simulation cell was filled with water, 0.9% NaCl, and counter ions [37]. The main simulation was then run with a particle mesh Ewald electrostatic potential, an 8.0 Å cutoff for nonbonded real space forces, a 4 fs time step, constrained hydrogen atoms, and at constant pressure and temperature (a temperature of 298 K and a pressure of 1 atm) [38]. YASARA uses the time averaged macroscopic temperature and pressure to resize the cell using the improved Berendsen thermostat and barostat [39]. The simulation system contained 37 Na ions, 43 Cl ions, and 158 lipid molecules. The system was solvated with 13,128 (MC4R WT); 13,132 (D122N); 13,142 (D126Y); 13,128 (S188L) water molecules. Analysis of the simulation trajectories were performed by the MD_analyze macro embedded in YASARA.

### 2.6. Principal Components Analysis (PCA)

The conformational flexibility and the collective motions of the MC4R wild type and mutants were analyzed using PCA [40]. Coordinates were superimposed onto a reference structure from which the positional covariance matrix of the atomic coordinates and its eigenvectors were calculated. Then, diagonal eigenvectors and the equivalent eigenvalues were derived based on the calculation and diagonalization of the covariant atomic fluctuation equation Each eigenvector was associated with an eigenvalue that represented the total mean square fluctuation of the system along the corresponding eigenvector. The mathematical details have been described previously [41].

## 3. Results and Discussion

Mutations in human MC4R are the most frequent cause of obesity. To understand the role of MC4R gene products in relation to obesity, we analyzed the single-nucleotide polymorphisms (SNPs) associated with this gene with the aim to understand the genetic variations and associated functional abnormalities. We curated 318 nonsynonymous mutations in MC4R from the Ensembl database and performed in silico mutation analysis using various pathogenic and stability analysis computational tools to predict the impact of the mutation on the MC4R receptor structure and function.

### 3.1. Predicting the Impact of nsSNPs of MC4R

**Figure 1:**
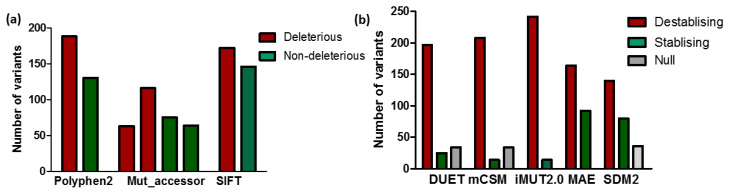
Effect of mutations by sequence and structure-based predictions**:** (**a**) deleterious (red) or non-deleterious (green) mutations in MC4R as predicted by sequence-based tools; (**b**) destabilizing (red), stabilizing (green), or null (grey) as predicted by structure-based analyses.

Computational analyses through a variety of tools were performed on MC4R single-nucleotide variants. Out of the 318 nsSNPs analyzed by SIFT, ~172 (54%) SNPs were predicted to be deleterious (tolerating index (TI) ≤ 0.05) and 146 SNPs were tolerated having a TI ≥ 0.05. Similarly, Polyphen2 predicted ~188 (59%) nsSNPs to be damaging and 130 as benign (Figure 1a). According to the Mutation Assessor (Mut_accessor) analysis, ~63 (19.8%) of mutations were found to be highly damaging, ~116 (36.4%) had a medium impact (both high and medium were deleterious, combined 56.2%), 75 (23.6%) had a low impact, and 64 (20.1%) SNPs were predicted as being neutral (Figure 1a). Overall, all of the three predictor models predicted over 50% of the mutations to be deleterious in nature. The use of multiple tools for analysis allowed for the generation of accurate data by purging false predictions. Despite the variability of the algorithms, the prediction outcomes of SIFT, Polyphen2, and Mut_accessor when the categories were binary were very similar and accounted for approximately 50–60% of the deleterious nsSNPs. 

### 3.2. Impact of Mutation on MC4R Stability

Most disease-associated nsSNPs affect protein stability. The effect of the mutations on the MC4R’s stability was investigated using structure-based computational tools, namely, DUET, mCSM, and SDM2, and stability was represented by the change in the Gibbs free energy (ΔG). The destabilizing mutations accounted for 197 (62%), 208 (65%), 242 (76%), 164 (52%), and 140 (44%) as detected by DUET, mCSM, iMUT2.0, MAE, and SDM2, respectively (Figure 1b).

### 3.3. Sequence Conservation Analysis

**Figure 2:**
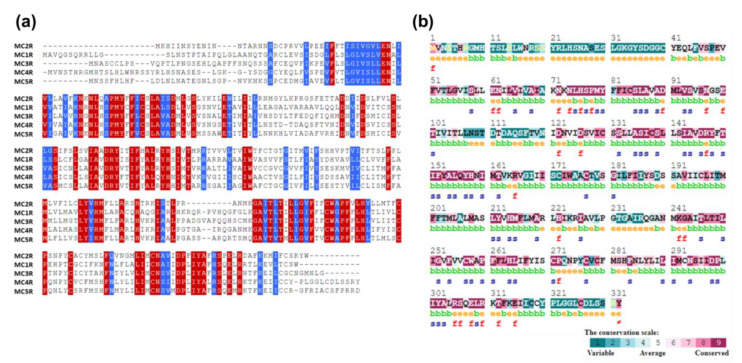
Sequence and structural conservation of MCRs**:** (**a**) Multiple sequence alignment of proteins in the MCR family, highlighting the high sequence conservation among the members. Identical amino acids are shown in red while similar amino acids are shown in blue. (**b**) Analysis of the evolutionary conservation of human MC4R sequence conservation using ConSurf (https://consurf.tau.ac.il/). The color-coding bar represents the coloring scheme of the conservation score: e—an exposed residue; b—a buried residue; f—a predicted functional residue (highly conserved and exposed); s—a predicted structural residue (highly conserved and buried).

The pattern of conservation of the amino acid residues is crucial to fully comprehending the evolutionary details with regards to its structure and function. Multiple sequence alignment of the MC4R family (i.e., MC2R, MC1R, MC3R, MC4R, and MC5R), as well as consurf analysis, indicated that the transmembrane helical regions were well conserved compared to the N- and C-terminal regions (Figure 2a). Active site residues, glutamate 100 (E100), aspartate 122 (D122), and D126, were highly conserved throughout the MC4R family (Figure 2b), highlighting their crucial role in ligand as well as metal binding. A disulfide bridge between cysteine 271 (C271) and C277, which is known to cause helical transition during MC4R activation, was conserved in the MC4R family, indicating a similar activation mechanism with the evolutionarily related GPCRs. A second disulfide bridge between C279 and C40 in the N-terminus was only conserved in MC4R and MC1R (C279 was conserved in all members, but C40 was present only in MC4R and MC1R) giving them their unique identity and agreeing with previous experimental studies on MC4R and MC1R, facilitating the stabilization of the ligand-binding pocket [42].

### 3.4. Structural Analysis of MC4R

**Figure 3:**
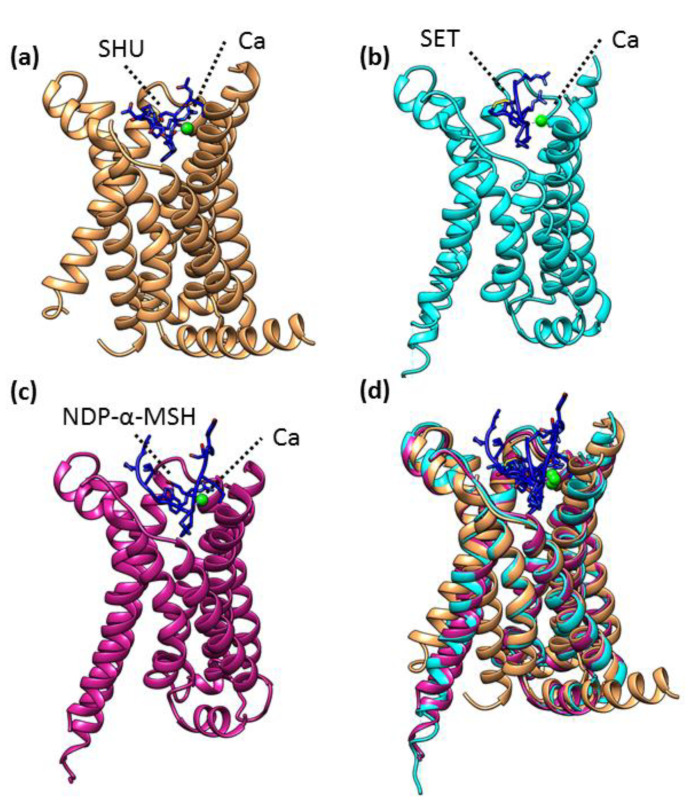
Structural details of melanocortin-4 receptor (MC4R): (**a**) The crystal structure of the MC4R in complex with SHU9119 (antagonist) (PDBID: 6W25). MC4R is shown in brown ribbons, SHU9119 in blue sticks, and Ca^2+^ in light green. (**b**) The Cryo-EM structure of MC4R in complex with setmelanotide (SET, agonist) (PDBID: 7AUE). MC4R is in cyan, setmelanotide (SET) is in blue, and Ca^2+^ is presented in light green. (**c**) Cryo-EM structures of MC4R in complex with NDP-α-MSH (agonist) (PDBID: 7PIV). MC4R is shown as magenta, NDP-α-MSH in blue, and Ca^2+^ is presented in light green. (**d**) Superposition of MC4R structures in complex with SHU9119 (antagonist), setmelanotide (SET, agonist), and NDP-α-MSH (agonist). MC4R is shown in brown, cyan, and magenta as bound to SHU9119, SET, and NDP-α-MSH, respectively. Calcium ions are shown as light green and all ligands as blue.

The first structure of human MC4R was solved by X-ray crystallography at a 2.8 angstrom resolution [30]. The structure was determined in complex with the antagonist SHU9119 and Ca^2+^ (PDBID: 6W25)^.^ MC4R is a classical seven-transmembrane helical protein containing a binding region to accommodate SHU9119 and a divalent cation Ca^2+^ (Figure 3a). Calcium ions are coordinated by the antagonist SHU9119 as well as by the MC4R residues, glutamate, and aspartate. Subsequently, the structure of MC4R was solved in complex with the agonist setmelanotide (a cyclic peptide approved for the treatment of obesity) by cryo-electron microscopy (cryo-EM) [29]. The active MC4R (MC4R–agonist) complex (PDBID: 7AUE) reveals the molecular mechanism including the molecular switch that initiates satiation signaling [29]. Similar to a crystal structure, Ca^2+^ ions occupied identical positions and were coordinated by both the agonist (i.e., setmelanotide) and the MC4R (Figure 3b). Recently, the MC4R complex with another agonist, NDP-α-MSH (an FDA-approved, high-affinity analog of the endogenous agonist α-MSH), was determined using cryo-EM (PDBID: 7PIV) (Figure 3c) and highlighted the crucial role of transmembrane helix 3 (TM3) in ligand-specific interactions and the role of a calcium ions in forming a link between ligands and TM2 and TM3 [43].

To understand the conformational changes that occurred among different MC4R complexes, we compared their structures by calculating the root mean square deviation (RMSD) of Cα atoms. The superposition of different MC4R structures showed RMSDs of approximately 0.82–1.58 Å for 230–258 Cα atoms, indicating that the overall conformations of all MC4R complex structures remained similar (Figure 3d). However, a large RMDS difference was observed between an inactive MC4R complex (i.e., MC4R–antagonist, PDBID: 6W25) and an active complex (i.e., MC4R–agonist, PDBID: 7AUE) (RMSDs of 1.58 Å), suggesting a major conformational change as reported earlier [29,31]. The conformational change in the outward displacement of TM6 differentiates the active and inactive forms of MC4R [29].

### 3.5. Mutations in the Calcium-Binding Site

**Figure 4:**
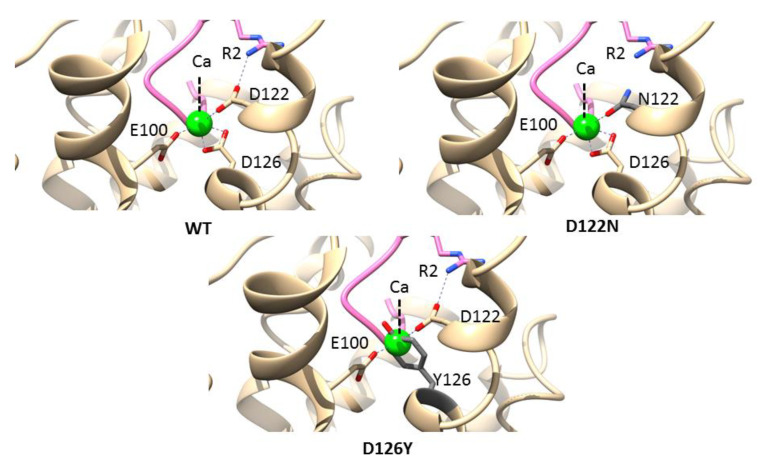
Mutations in the calcium-binding site. A structural view of the calcium ion-binding pocket (PDBID: 7AUE), highlighting the interactions between the calcium ion (green) and MC4R (tan). Residues from helices II and III of MC4R, especially the negative charges from glutamate and aspartate, coordinate with calcium (**left panel**). A mutation at position 122 from aspartate to asparagine (D122N) would hamper electrostatic interactions with the ligand (**right panel**). Similarly, a mutation at position 126 from aspartate to tyrosine (D126Y) would collapse the active site structure due to the bulkier aromatic sidechain of tyrosine, which sterically clash with calcium (**lower panel**).

Ca^2+^ is a critical cofactor for MC4R and helps in binding to the endogenous agonist α-MSH. The calcium ion was coordinated by three negatively charged residues of MC4R, namely, E100, D122, and D126 (Figure 4, left panel). Two naturally occurring mutations related to obesity were reported in this pocket: D122N and D126Y [44,45]. The mutations were introduced into the MC4R complex structure, and the interactions were analyzed using the structural analysis software program UCSF Chimera [46]. The D122 coordinates with calcium as well as with the agonist/antagonist arginine (R) and is crucial for maintaining the active site pocket. Two carboxy oxygens of aspartate were utilized: one to coordinate calcium and the other to interact with the terminal amine of arginine. Mutations in asparagine (D122N) would hamper a critical electrostatic interaction with the agonist/antagonist due to the similar charge repulsions (Figure 4, right panel). Similarly, D126 interacts with both calcium and the agonist/antagonist, and a mutation in tyrosine (D126Y) would collapse the active site structure due to the bulkier aromatic sidechain of tyrosine, which sterically clashes with calcium (Figure 4, lower panel). These results are in accordance with previous report that D122N mutant efficacy decreases for α-MSH and setmelanotide under physiological Ca^2+^ concentrations, and the D126Y mutant resulted in the complete abolishment of receptor function [47].

### 3.6. Mutations in the Agonist/Antagonist-Binding Pocket

**Figure 5:**
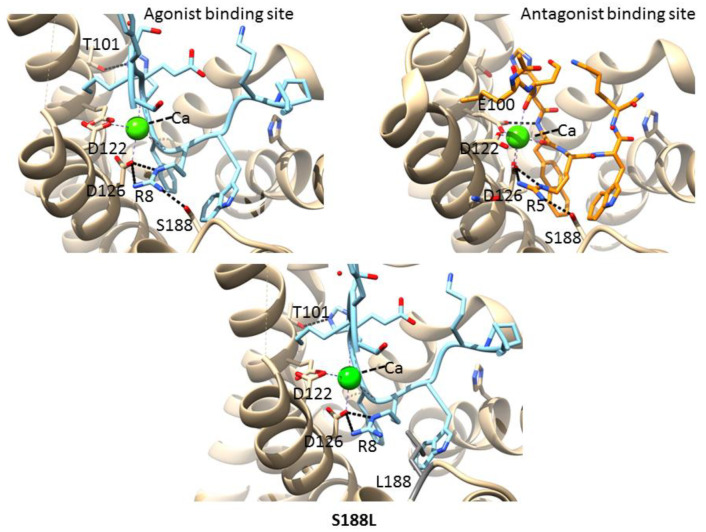
Mutation in the agonist/antagonist pocket. A structural view of the active site pocket (ligand as well as metal ion-binding site). Expanded view of the agonist (PDBID: 7PIV) (**left panel**) and antagonist (PDBID: 6W25) (**right panel**) binding pocket showing the interaction network among MC4R (tan), agonist/antagonist (cyan/brown), and calcium ion (green). The interactions between the metal ion, MC4R, and the agonist/antagonist are represented by the solid black lines. A mutation at position 188 from serine to leucine (S188L) would disturb the hydrogen bond network (**lower panel**).

The MC4R complex structures (agonist/antagonist bound) show a binding pocket containing agonist/antagonist and a divalent cation, calcium. The agonist/antagonist engages MC4R through extensive van der Waals, hydrophobic, and polar interactions, with residues from TMs as well as extracellular loops [43,47,48]. Aspartate (D122 and D126) of MC4R formed part of an agonist/antagonist and Ca^2+^-binding network (Figure 5, left and right panels). Both agonist and antagonist share a core central amino acid motif HxRW (H—histidine, R—arginine, and W—tryptophan). This motif of agonist/antagonist interacts with MC4R through multiple bonds, most noticeably through one salt bridge (R8 and R5 of the agonist and antagonist, respectively, to D126 of MC4R) and hydrogen bond between R8 and R5 of the agonist and antagonist, respectively, with S188 (present in an extracellular loop) of MC4R (Figure 5, left and right panels). A natural variant at position 188 is known (S188L). The change in the negative charge of serine to a hydrophobic residue, leucine, would destabilize the region by not forming a hydrogen bond to the agonist/antagonist core motif (43,47,48]. Aspartate (D122 and D126) of MC4R formed part of an agonist/antagonist and Ca^2+^-binding network (Figure 5, lower panel). As given in Table 1, the S188L mutation had a greater destabilizing effect compared to the wild type. All three mutations induced instability in MC4R (Table 1).

### 3.7. Dynamics of the Wild-Type and Selected Mutant Complexes

**Figure 6:**
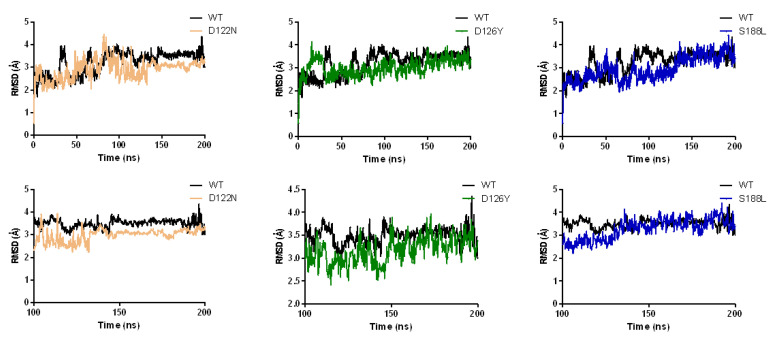
Analysis of the molecular dynamic trajectories of 200 ns by root mean square deviation (RMSD) plots of wild-type and mutant MC4R complex structures. The RMSD plots show the deviation of mutants compared to the wild type during the simulation.

To gain insight into the effects of three mutations (i.e., D122N, D126Y, and S188L) on the MC4R complex’s structure, molecular dynamics simulations were carried out for 200 ns. The resultant trajectory files were used for evaluating the structural changes observed between the wild-type and mutant forms of MC4R. The dynamic characteristics of the wild type and mutants were monitored by observing the root mean square deviation (RMSD) trajectories of the Cα atoms. The wild type showed changes in structure from the start of the simulation to 200 ns. The initial RMSD was 2–3 Å, increased gradually up to 90 ns (from 2–3 to 3.5 Å) and remained the same until the end of the simulation (Figure 6). The D122N mutant’s RMSD values were similar to the wild type until 100 ns, after which it showed a higher stability with a lower RMSD (RMSD decreased to 3 Å). Overall, similar but distinct RMSD ranges were observed for the wild-type and the mutant MC4R. The average RMSD values and the convergence of all the simulations suggest that the simulations produced stable trajectories, providing a suitable basis for further analyses. 

**Figure 7:**
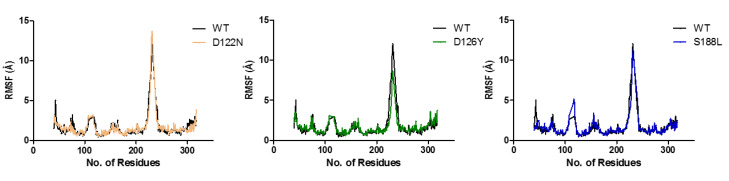
Root Mean Square Fluctuation (RMSF) analysis of the native and mutant forms of MC4R. RMSF plot shows the carbon backbone atom fluctuations in wild type and mutants.

**Figure 8:**
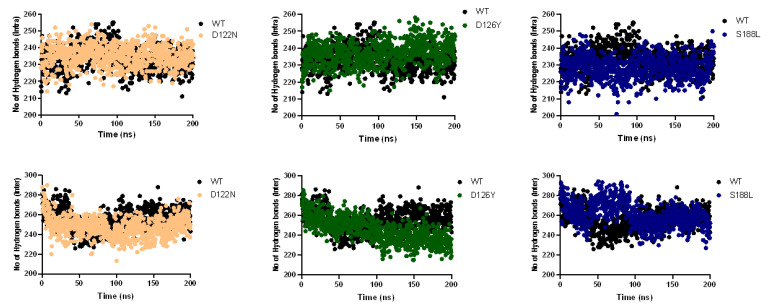
Hydrogen bond (intra- as well as intermolecular) analyses during a 200 ns simulation. The plots reveal the deviations in the hydrogen bond formation of wild-type MC4R and the mutants during the 200 ns simulation.

The root mean square fluctuations (RMSFs) of the Cα were calculated from the MD simulations to find the local fluctuations in the MC4R complex’s structure due to the mutations (i.e., D122N, D126Y, and S188L) (Figure 7). All the mutants showed overall similar fluctuation behaviors compared to the wild type. In the case of S188L, an increase in internal fluctuation was observed at the active site region between amino acid residues 100 and 130, indicating that the mutation affected protein conformation, leading to an increased flexibility of residues in that region while maintaining the wild-type-like flexibility of the residues in other regions. The fluctuation observed may be due to the fact of its crucial interaction with ligands. S188-R (agonist/antagonist)-D126 formed a bridge connecting the active site to the S188 region. The ligand acts as a bridge to connect S188 to the active site residue D126, and the mutation disrupted this interaction, which may have an effect on active site fluctuation. We also performed intramolecular hydrogen bond analysis to predict any change in the number of hydrogen bonds formed during the simulation. The results of the hydrogen bond analysis of the wild-type and the mutant protein performed with respect to the time indicated that the mutant S188L had significantly fewer hydrogen bond formations during the entire simulation compared to the wild-type MC4R. The hydrogen bond analysis predicted no noticeable change in the number of intramolecular hydrogen bonds during the simulation in the case of the D122N and D126Y mutants (Figure 8).

**Figure 9:**
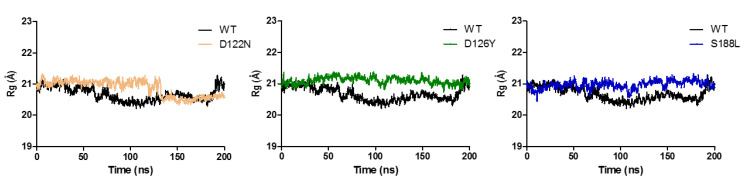
Analysis of the molecular dynamic trajectories over 200 ns by radius of gyration (Rg) plots of wild-type and mutant MC4R complex structures. The plots highlight the change in the Rg of the mutant forms compared to the wild type during a 200 ns simulation.

Protein compactness was measured through the radius of gyration. A higher Rg values depict less compactness (i.e., more unfolded) with conformational entropy, while low Rg values explain high compactness with more stability in the structure (i.e., more folded). The wild-type Rg was found to be almost stable while maintaining an Rg score of 21 Å (Figure 9). Compared to the wild type, the mutants had higher Rg values, indicating that the mutants led to disturbances in the compactness of the MC4R complex. 

**Figure 10:**
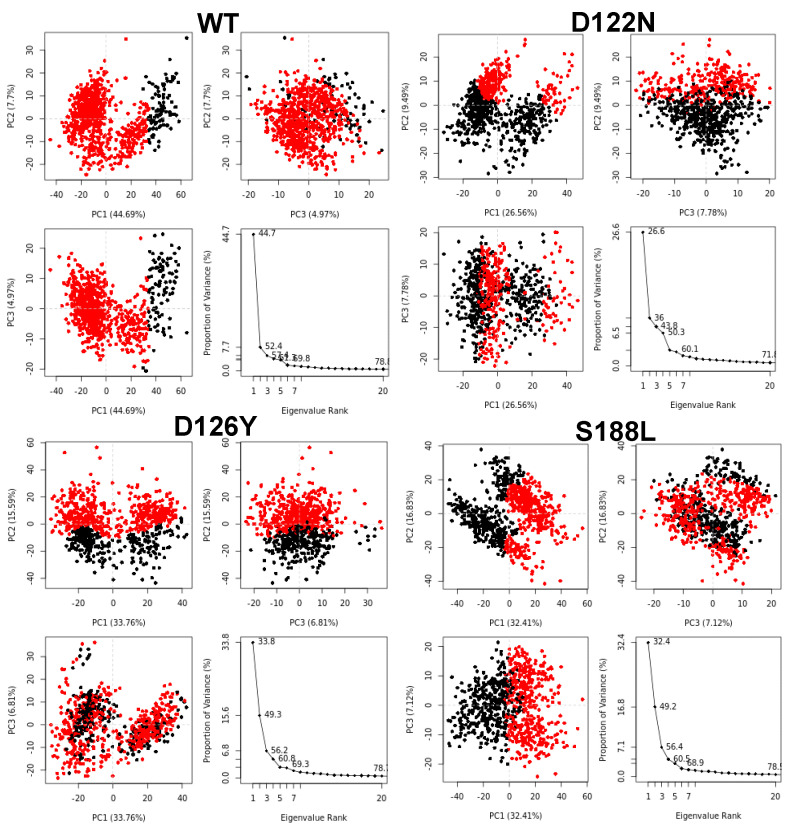
Principal component analysis (PCA) of wild-type and mutant MC4R complexes. The projections of the simulated trajectories on the first three eigenvectors, based on complex dominant motion, were extracted and plotted. Each dot represents one conformation of the protein along the *x*- and *y*-axes. Red and black represent clusters of movements.

Additionally, PCA was performed to analyze the dominant motions in the MC4R wild-type and mutant complexes. It captures the combined dominant motions through several eigenvectors and can be applied to any system, permitting the study of the influence of any varying parameters by lessening the collective motions’ complexity [49,50,51]. PCA indicated that the first three PCs accounted for 57.4%, 43.8%, 56.2%, and 56.4% of the variance in the motion observed in the trajectories of WT, D122N, D126Y, and S188L, respectively (Figure 10). The magnitude of PC1 was the highest in the WT and decreased by mutations (a first PCA of 44.7%, 26.6%, 33.8%, and 32.4% for the WT D122N, D126Y, and S188L, respectively). All of the MC4R complexes showed distinct clustering, suggesting that it may undergo a periodic shift in its conformation to reorient its transmembrane domains. The highest PC1 in the WT structure indicated conformational changes required to perform the function, while mutated forms endured these conformational changes due to the mutation.

Unlike other GPCRs, the role of calcium in the function of MC4R has been recently established. Our results, in concordance with these studies, provide evidence to establish the crucial role of important amino acids in the calcium-, agonist-, and antagonist-binding pockets. Mutations in these residues, particularly their replacement into certain residues, proved to be deleterious/highly destabilizing (i.e., D122N, D126Y, and S188L) in our study as evident from our in silico data analysis and molecular simulation studies. These mutations were either calcium coordinating residues or interacted with an agonist/antagonist. D122N decreased the efficacy for alpha-MSH and setmelanotide [47,48] D126Y abolished the receptor function completely [48]. As discussed earlier, these mutations cause changes in the interaction in the active site pockets with respect to ion or hydrogen bonding or steric interaction, destabilizing the overall structure or function of the protein. S188L exerted its effect on the activity of MC4R by interacting with the substrate.

## 4. Conclusions

Genetic variations, including nonsynonymous mutations in MC4R, are a leading cause of obesity. Ca^2+^ is a cofactor and integral part of the ligand-binding pocket. The MC4R ligand (agonist/antagonist)-binding vestibule was adapted to integrate signals from ligands of various sizes and to induce specific cellular responses via different G protein signaling pathways. Mutations in this pocket, either involving Ca^2+^ or agonist/antagonist binding, had adverse effects on the cellular responses and the functioning of the MC4R receptor. Active site mutations were examined by MD simulations to elucidate the effects of the mutations on the structure and stability of the MC4R receptor. The effects of the D122N, D126Y, and S188L mutations (also comprehensive in silico predictive mutational analysis of all the mutations reported) on protein dynamics using 200 ns simulations revealed the extent of the flexibility of the complex structure. Our work provides additional insight into the contribution of genetic variations in MC4R in terms of its pathological effect as well as on protein stability. Ligand specificity, the role of mutants crucial in the activation and/or inhibition of the receptor, as well as their role in altering the complex structure were investigated. As detailed and precise understanding will, we believe, help in future endeavors to develop well-designed therapeutic strategies to fight obesity and related diseases.

## Figures and Tables

**Table 1:** Predicted protein stability scores of the mutants. The I-Mutant2.0 and mCSM-membrane predicted stability effects upon mutation. ΔΔG < 0 (values of energies are given in kcal/mol) indicates a reduction in protein stability.

S. No.	Variants	Predicted ΔΔG (I-Mutant2.0)	Predicted ΔΔG (mCSM-Membrane)	Outcome
1	WT	00	00	-
2	D122N	−2.64	−0.575	Highly destabilizing
3	D126Y	−2.41	−0.913	Highly destabilizing
4	S188L	−2.20	−0.648	Highly destabilizing

## Data Availability

Not applicable.

## Sample Availability

Samples of the compounds are not available from the authors.

## References

[1] Krashes M.J., Lowell B.B., Garfield A.S. (2016). Melanocortin-4 Receptor-Regulated Energy Homeostasis. Nat. Neurosci..

[2] Fatima M.T., Ahmed I., Fakhro K.A., Akil A.S.A.-S. (2022). Melanocortin-4 Receptor Complexity in Energy Homeostasis, Obesity and Drug Development Strategies. Diabetes Obes. Metab..

[3] Baldini G., Phelan K.D. (2019). The Melanocortin Pathway and Control of Appetite-Progress and Therapeutic Implications. J. Endocrinol..

[4] Huvenne H., Dubern B., Clément K., Poitou C. (2016). Rare Genetic Forms of Obesity: Clinical Approach and Current Treatments in 2016. Obes. Facts.

[5] Gonçalves J.P.L., Palmer D., Meldal M. (2018). MC4R Agonists: Structural Overview on Antiobesity Therapeutics. Trends Pharmacol. Sci..

[6] Lotta L.A., Mokrosiński J., de Oliveira E.M., Li C., Sharp S.J., Luan J., Brouwers B., Ayinampudi V., Bowker N., Kerrison N. (2019). Human Gain-of-Function MC4R Variants Show Signaling Bias and Protect against Obesity. Cell.

[7] Tao Y.-X. (2010). The Melanocortin-4 Receptor: Physiology, Pharmacology, and Pathophysiology. Endocr. Rev..

[8] Kühnen P., Krude H., Biebermann H. (2019). Melanocortin-4 Receptor Signalling: Importance for Weight Regulation and Obesity Treatment. Trends Mol. Med..

[9] Namjou B., Stanaway I.B., Lingren T., Mentch F.D., Benoit B., Dikilitas O., Niu X., Shang N., Shoemaker A.H., Carey D.J. (2021). Evaluation of the MC4R Gene across EMERGE Network Identifies Many Unreported Obesity-Associated Variants. Int. J. Obes..

[10] Aykut A., Özen S., Gökşen D., Ata A., Onay H., Atik T., Darcan Ş., Özkinay F. (2020). Melanocortin 4 Receptor (MC4R) Gene Variants in Children and Adolescents Having Familial Early-Onset Obesity: Genetic and Clinical Characteristics. Eur. J. Pediatr..

[11] Lubrano-Berthelier C., Cavazos M., Dubern B., Shapiro A., Stunff C.L.E., Zhang S., Picart F., Govaerts C., Froguel P., Bougneres P. (2003). Molecular Genetics of Human Obesity-Associated MC4R Mutations. Ann. N. Y. Acad. Sci..

[12] MacKenzie R.G. (2006). Obesity-Associated Mutations in the Human Melanocortin-4 Receptor Gene. Peptides.

[13] Huszar D., Lynch C.A., Fairchild-Huntress V., Dunmore J.H., Fang Q., Berkemeier L.R., Gu W., Kesterson R.A., Boston B.A., Cone R.D. (1997). Targeted Disruption of the Melanocortin-4 Receptor Results in Obesity in Mice. Cell.

[14] Büch T.R.H., Heling D., Damm E., Gudermann T., Breit A. (2009). Pertussis Toxin-Sensitive Signaling of Melanocortin-4 Receptors in Hypothalamic GT1-7 Cells Defines Agouti-Related Protein as a Biased Agonist. J. Biol. Chem..

[15] Kühnen P., Clément K., Wiegand S., Blankenstein O., Gottesdiener K., Martini L.L., Mai K., Blume-Peytavi U., Grüters A., Krude H. (2016). Proopiomelanocortin Deficiency Treated with a Melanocortin-4 Receptor Agonist. N. Engl. J. Med..

[16] Clément K., Biebermann H., Farooqi I.S., Van der Ploeg L., Wolters B., Poitou C., Puder L., Fiedorek F., Gottesdiener K., Kleinau G. (2018). MC4R Agonism Promotes Durable Weight Loss in Patients with Leptin Receptor Deficiency. Nat. Med..

[17] (2021). UniProt Consortium UniProt: The Universal Protein Knowledgebase in 2021. Nucleic Acids Res..

[18] Hubbard T., Barker D., Birney E., Cameron G., Chen Y., Clark L., Cox T., Cuff J., Curwen V., Down T. (2002). The Ensembl Genome Database Project. Nucleic Acids Res..

[19] Kumar P., Henikoff S., Ng P.C. (2009). Predicting the Effects of Coding Non-Synonymous Variants on Protein Function Using the SIFT Algorithm. Nat. Protoc..

[20] Adzhubei I.A., Schmidt S., Peshkin L., Ramensky V.E., Gerasimova A., Bork P., Kondrashov A.S., Sunyaev S.R. (2010). A Method and Server for Predicting Damaging Missense Mutations. Nat. Methods.

[21] Reva B., Antipin Y., Sander C. (2011). Predicting the Functional Impact of Protein Mutations: Application to Cancer Genomics. Nucleic Acids Res..

[22] Pires D.E.V., Ascher D.B., Blundell T.L. (2014). MCSM: Predicting the Effects of Mutations in Proteins Using Graph-Based Signatures. Bioinformatics.

[23] Kumar M.D.S., Bava K.A., Gromiha M.M., Prabakaran P., Kitajima K., Uedaira H., Sarai A. (2006). ProTherm and ProNIT: Thermodynamic Databases for Proteins and Protein-Nucleic Acid Interactions. Nucleic Acids Res..

[24] Moal I.H., Fernández-Recio J. (2012). SKEMPI: A Structural Kinetic and Energetic Database of Mutant Protein Interactions and Its Use in Empirical Models. Bioinformatics.

[25] Pandurangan A.P., Ochoa-Montaño B., Ascher D.B., Blundell T.L. (2017). SDM: A Server for Predicting Effects of Mutations on Protein Stability. Nucleic Acids Res..

[26] Pires D.E.V., Ascher D.B., Blundell T.L. (2014). DUET: A Server for Predicting Effects of Mutations on Protein Stability Using an Integrated Computational Approach. Nucleic Acids Res..

[27] Capriotti E., Fariselli P., Casadio R. (2005). I-Mutant2.0: Predicting Stability Changes upon Mutation from the Protein Sequence or Structure. Nucleic Acids Res..

[28] Laimer J., Hiebl-Flach J., Lengauer D., Lackner P. (2016). MAESTROweb: A Web Server for Structure-Based Protein Stability Prediction. Bioinformatics.

[29] Madeira F., Park Y.M., Lee J., Buso N., Gur T., Madhusoodanan N., Basutkar P., Tivey A.R.N., Potter S.C., Finn R.D. (2019). The EMBL-EBI Search and Sequence Analysis Tools APIs in 2019. Nucleic Acids Res..

[30] Ashkenazy H., Erez E., Martz E., Pupko T., Ben-Tal N. (2010). ConSurf 2010: Calculating Evolutionary Conservation in Sequence and Structure of Proteins and Nucleic Acids. Nucleic Acids Res..

[31] Krieger E., Vriend G. (2014). YASARA View—Molecular Graphics for All Devices—From Smartphones to Workstations. Bioinformatics.

[32] Maier J.A., Martinez C., Kasavajhala K., Wickstrom L., Hauser K.E., Simmerling C. (2015). Ff14SB: Improving the Accuracy of Protein Side Chain and Backbone Parameters from Ff99SB. J. Chem. Theory Comput..

[33] Gould I.R., Skjevik A.A., Dickson C.J., Madej B.D., Walker R.C. (2018). Lipid17: A Comprehensive AMBER Force Field for the Simulation of Zwitterionic and Anionic Lipids. Manuscr. Prep..

[34] Wang J., Wolf R.M., Caldwell J.W., Kollman P.A., Case D.A. (2004). Development and Testing of a General Amber Force Field. J. Comput. Chem..

[35] Jakalian A., Jack D.B., Bayly C.I. (2002). Fast, Efficient Generation of High-Quality Atomic Charges. AM1-BCC Model: II. Parameterization and Validation. J. Comput. Chem..

[36] Krieger E., Nielsen J.E., Spronk C.A.E.M., Vriend G. (2006). Fast Empirical PKa Prediction by Ewald Summation. J. Mol. Graph. Model..

[37] Krieger E., Darden T., Nabuurs S.B., Finkelstein A., Vriend G. (2004). Making Optimal Use of Empirical Energy Functions: Force-Field Parameterization in Crystal Space. Proteins.

[38] Krieger E., Vriend G. (2015). New Ways to Boost Molecular Dynamics Simulations. J. Comput. Chem..

[39] Berendsen H.J.C., Postma J.P.M., van Gunsteren W.F., DiNola A., Haak J.R. (1984). Molecular Dynamics with Coupling to an External Bath. J. Chem. Phys..

[40] Ichiye T., Karplus M. (1991). Collective Motions in Proteins: A Covariance Analysis of Atomic Fluctuations in Molecular Dynamics and Normal Mode Simulations. Proteins.

[41] Shlens J. (2014). A Tutorial on Principal Component Analysis. arXiv.

[42] Chai B.-X., Pogozheva I.D., Lai Y.-M., Li J.-Y., Neubig R.R., Mosberg H.I., Gantz I. (2005). Receptor-Antagonist Interactions in the Complexes of Agouti and Agouti-Related Protein with Human Melanocortin 1 and 4 Receptors. Biochemistry.

[43] Heyder N.A., Kleinau G., Speck D., Schmidt A., Paisdzior S., Szczepek M., Bauer B., Koch A., Gallandi M., Kwiatkowski D. (2021). Structures of Active Melanocortin-4 Receptor-Gs-Protein Complexes with NDP-α-MSH and Setmelanotide. Cell Res..

[44] Collet T.-H., Dubern B., Mokrosinski J., Connors H., Keogh J.M., de Oliveira E.M., Henning E., Poitou-Bernert C., Oppert J.-M., Tounian P. (2017). Evaluation of a Melanocortin-4 Receptor (MC4R) Agonist (Setmelanotide) in MC4R Deficiency. Mol. Metab..

[45] Stutzmann F., Tan K., Vatin V., Dina C., Jouret B., Tichet J., Balkau B., Potoczna N., Horber F., O’Rahilly S. (2008). Prevalence of Melanocortin-4 Receptor Deficiency in Europeans and Their Age-Dependent Penetrance in Multigenerational Pedigrees. Diabetes.

[46] Pettersen E.F., Goddard T.D., Huang C.C., Couch G.S., Greenblatt D.M., Meng E.C., Ferrin T.E. (2004). UCSF Chimera—A Visualization System for Exploratory Research and Analysis. J. Comput. Chem..

[47] Israeli H., Degtjarik O., Fierro F., Chunilal V., Gill A.K., Roth N.J., Botta J., Prabahar V., Peleg Y., Chan L.F. (2021). Structure Reveals the Activation Mechanism of the MC4 Receptor to Initiate Satiation Signaling. Science.

[48] Yu J., Gimenez L.E., Hernandez C.C., Wu Y., Wein A.H., Han G.W., McClary K., Mittal S.R., Burdsall K., Stauch B. (2020). Determination of the Melanocortin-4 Receptor Structure Identifies Ca2+ as a Cofactor for Ligand Binding. Science.

[49] Amadei A., Linssen A.B., Berendsen H.J. (1993). Essential Dynamics of Proteins. Proteins.

[50] Kitao A., Go N. (1999). Investigating Protein Dynamics in Collective Coordinate Space. Curr. Opin. Struct. Biol..

[51] (1992). García Large-Amplitude Nonlinear Motions in Proteins. Phys. Rev. Lett..

